# Fosgonimeton attenuates amyloid‐β‐induced autophagic impairments in primary cortical neurons

**DOI:** 10.1002/alz.089252

**Published:** 2025-01-09

**Authors:** Sherif M Reda, Wei Wu, Andrée‐Anne Berthiaume, Sharay E. Setti, Robert W Taylor, Jewel L Johnston, Kevin J. Church

**Affiliations:** ^1^ Athira Pharma, Inc., Bothell, WA USA

## Abstract

**Background:**

Accumulating evidence highlights impairment of autophagy as a key pathological feature of neurodegenerative diseases including Alzheimer’s disease (AD). Autophagy is a highly dynamic, lysosome‐based degradation process that promotes the clearance of degenerative factors to maintain cellular functions, preserve metabolic integrity, and ensure survival. Impaired autophagic function leads to the abnormal accumulation of autophagic vesicles (i.e., autophagosomes, lysosomes), build‐up of toxic protein aggregates, dysregulated homeostasis, synaptic dysfunction, and neurotoxicity. We have previously demonstrated that fosgonimeton, an investigational small‐molecule positive modulator of the neurotrophic hepatocyte growth factor (HGF) system, is neuroprotective and reduces pTau levels in amyloid‐beta (Aβ)‐driven preclinical models of AD. Herein, we investigate the effect of fosgonimeton on autophagic function in cortical neurons following Aβ injury to further characterize the neuroprotective effects of fosgonimeton treatment.

**Method:**

To evaluate potential effects on autophagic function, primary rat cortical neurons were treated with the active metabolite of fosgonimeton (fosgo‐AM), challenged with Aβ1‐42 (15 µM) for 24 hours, and immunostained for microtubule‐associated protein‐2 (neuronal marker), phospho‐tau, LC3 (autophagosome marker), and LAMP2 (lysosomal marker). Immunofluorescence analyses were used to determine neuronal survival, neurite network integrity, pTau levels, and expression/co‐localization of LC3 and LAMP2. Beclin‐1 (autophagy initiator) expression was also measured via western blot.

**Result:**

Fosgo‐AM treatment promoted neuronal survival, preserved network connectivity, and reduced levels of pTau following Aβ1‐42 challenge. Application of Aβ1‐42 significantly decreased the expression of Beclin‐1, led to the abnormal accumulation of autophagic vesicles (LC3, LAMP2, and LC3‐LAMP2 co‐localization), and increased the co‐localization of pTau within LC3‐autophagic vesicles, all of which are indicative of impaired autophagic function. Such effects were significantly attenuated in the presence of fosgo‐AM, suggesting that the neuroprotective effects of fosgonimeton are associated with improved autophagic function.

**Conclusion:**

The data presented demonstrate the ability of fosgonimeton to potentially address autophagic impairment in AD, which may have important implications regarding the attenuation of pTau pathology. These effects of fosgonimeton, in addition to the neurotrophic and neuroprotective effects previously demonstrated in preclinical models, highlight the therapeutic potential of the HGF system for neurodegenerative disorders, wherein autophagic impairment is a key pathological hallmark.

